# β-cell 14-3-3-ζ and sex are key regulators of α-cell form and function

**DOI:** 10.1210/endocr/bqag098

**Published:** 2026-08-26

**Authors:** Rahaf Shishani, Rosalinda Moreno, Madelynn E Agabao-Tucker, Sofie E Dinglasan, Jessica Miranda Bustamante, Emily Eddy, Anna Jackson, Melanie A Reuter, Mara K Hon, Mark O Huising, Gareth E Lim, Bethany P Cummings

**Affiliations:** Division of Foregut, Metabolic, and General Surgery, Department of Surgery, Center for Alimentary and Metabolic Sciences, School of Medicine, University of California—Davis, Sacramento, CA 95817, USA; Department of Molecular Biosciences, School of Veterinary Medicine, University of California—Davis, Davis, CA 95616, USA; Division of Foregut, Metabolic, and General Surgery, Department of Surgery, Center for Alimentary and Metabolic Sciences, School of Medicine, University of California—Davis, Sacramento, CA 95817, USA; Department of Molecular Biosciences, School of Veterinary Medicine, University of California—Davis, Davis, CA 95616, USA; Division of Foregut, Metabolic, and General Surgery, Department of Surgery, Center for Alimentary and Metabolic Sciences, School of Medicine, University of California—Davis, Sacramento, CA 95817, USA; Department of Molecular Biosciences, School of Veterinary Medicine, University of California—Davis, Davis, CA 95616, USA; Division of Foregut, Metabolic, and General Surgery, Department of Surgery, Center for Alimentary and Metabolic Sciences, School of Medicine, University of California—Davis, Sacramento, CA 95817, USA; Department of Molecular Biosciences, School of Veterinary Medicine, University of California—Davis, Davis, CA 95616, USA; Division of Foregut, Metabolic, and General Surgery, Department of Surgery, Center for Alimentary and Metabolic Sciences, School of Medicine, University of California—Davis, Sacramento, CA 95817, USA; Department of Molecular Biosciences, School of Veterinary Medicine, University of California—Davis, Davis, CA 95616, USA; Division of Foregut, Metabolic, and General Surgery, Department of Surgery, Center for Alimentary and Metabolic Sciences, School of Medicine, University of California—Davis, Sacramento, CA 95817, USA; Department of Molecular Biosciences, School of Veterinary Medicine, University of California—Davis, Davis, CA 95616, USA; Division of Foregut, Metabolic, and General Surgery, Department of Surgery, Center for Alimentary and Metabolic Sciences, School of Medicine, University of California—Davis, Sacramento, CA 95817, USA; Department of Molecular Biosciences, School of Veterinary Medicine, University of California—Davis, Davis, CA 95616, USA; Division of Foregut, Metabolic, and General Surgery, Department of Surgery, Center for Alimentary and Metabolic Sciences, School of Medicine, University of California—Davis, Sacramento, CA 95817, USA; Department of Molecular Biosciences, School of Veterinary Medicine, University of California—Davis, Davis, CA 95616, USA; Division of Foregut, Metabolic, and General Surgery, Department of Surgery, Center for Alimentary and Metabolic Sciences, School of Medicine, University of California—Davis, Sacramento, CA 95817, USA; Department of Molecular Biosciences, School of Veterinary Medicine, University of California—Davis, Davis, CA 95616, USA; Department of Neurobiology, Physiology and Behavior, College of Biological Sciences, University of California—Davis, Davis, CA 95616, USA; Department of Physiology and Membrane Biology, School of Medicine, University of California—Davis, Davis, CA 95616, USA; Department of Medicine, Université de Montréal, Montréal, QC H3T 1J4, Canada; Cardiometabolic Axis, Centre de Recherche du Centre hospitalier de l'Université de Montréal (CRCHUM), Montréal, QC H2X 049, Canada; Division of Foregut, Metabolic, and General Surgery, Department of Surgery, Center for Alimentary and Metabolic Sciences, School of Medicine, University of California—Davis, Sacramento, CA 95817, USA; Department of Molecular Biosciences, School of Veterinary Medicine, University of California—Davis, Davis, CA 95616, USA

**Keywords:** 14-3-3-ζ, GLP-1, α-cell

## Abstract

α-cells exhibit heterogeneity in their hormone production, whereby distinct α-cell subpopulations can be induced to produce active glucagon-like peptide-1 (GLP-1). However, the mechanisms that govern α-cell GLP-1 production remain poorly defined and whether these differ between sex is unknown. We previously identified 14-3-3-ζ as an indirect negative regulator of α-cell GLP-1 production in vitro. Therefore, we tested the hypothesis that β-cell 14-3-3-ζ ablation exerts sex-dependent protection against metabolic disease through alteration of the α-cell endocrine profile. Male and female mice with targeted manipulation of GLP-1 production in α-cells and 14-3-3-ζ expression in β-cells were studied. Further, publicly available human pancreas data were analyzed to identify sex-dependent differences in islet cell populations. Loss of α-cell-derived GLP-1 impaired β-cell compensation in response to high-fat diet-induced metabolic stress, and β-cell 14-3-3-ζ ablation improved glucose tolerance during a mixed-meal tolerance test in male, but not female, mice. Conversely, overexpression of 14-3-3-ζ blunted the effect of a GLP-1 receptor agonist to promote insulin secretion in male, but not female, mice. Further, β-cell 14-3-3-ζ ablation increased α-cell abundance in male mice while enhancing α-cell GLP-1 production and secretion in both sexes. Finally, α-cell numbers were higher in females compared to males in both mouse and human islets, and α-cell GLP-1 production was higher in females compared to male mice. Together, these findings identify β-cell 14-3-3-ζ as an upstream constraint on α-cell GLP-1 production and reveal sex-dependent regulation of α-cell abundance, establishing β-cell 14-3-3-ζ and biological sex as key determinants of α-cell GLP-1 production.

Islet hormone secretion is tightly regulated by islet cell crosstalk, with increasing appreciation for the contribution of α-cell proglucagon-derived peptides in supporting β-cell insulin secretion (1-8). This intra-islet communication is essential for maintaining glucose homeostasis and is increasingly recognized as a dynamic process influenced by metabolic cues and paracrine signaling mechanisms. However, there remain gaps in our understanding of the mechanisms by which α-cells support β-cell function. In particular, how α-cell endocrine heterogeneity influences α-cell to β-cell crosstalk is poorly defined.

Classically, glucagon protects against hypoglycemia by increasing blood glucose concentrations through increased hepatic glucose production. In addition to these systemic effects, glucagon also acts locally within the islet to support insulin secretion by directly activating β-cell glucagon and glucagon-like peptide 1 (GLP-1) receptors (9-11). Consistent with this intra-islet role, genetic and pharmacologic studies of disruption of signaling through either receptor reveal impaired insulin secretion and altered glucose tolerance (1, 4). However, α-cells exhibit heterogeneity in their transcriptome, electrophysiological properties, and endocrine profile—defined as the hormonal output of α-cells. While α-cells typically produce glucagon, under certain conditions, α-cells can produce GLP-1. Although GLP-1 is classically recognized as an incretin hormone secreted from enteroendocrine L-cells in the gut in response to nutrient intake (12, 13), the capacity of the pancreatic α-cell to produce GLP-1 has garnered increasing recognition, particularly given its well-established role in enhancing glucose-stimulated insulin secretion (GSIS) (14). GLP-1 was thought to exert its incretin effect primarily after its secretion from the L cell, after which it circulates systemically to activate its receptor on the β-cell. However, the low postprandial levels of circulating active GLP-1 and its rapid enzymatic degradation by dipeptidyl peptidase-4 (DPP-4) (15, 16) have raised questions regarding the sufficiency of the gut as the sole source of GLP-1.

These findings have prompted increased interest in the α-cell as a site of GLP-1 production. The capacity of the α-cell to make GLP-1 is due to tissue-specific posttranslational processing of proglucagon by prohormone convertases (PC). PC2 cleaves proglucagon into glucagon, whereas PC1/3 cleaves proglucagon to yield GLP-1. α-cells typically express PC2 but can express PC1/3 and generate active GLP-1 under conditions such as obesity and type 2 diabetes (1-8, 17-23). However, the physiological regulation of α-cell GLP-1 and the mechanisms underlying the shift in α-cell hormone profile remain poorly understood. GLP-1R agonists demonstrate substantial efficacy in the treatment of type 2 diabetes. Still, exogenous GLP-1 often causes adverse effects that limit long-term adherence (24). Therefore, it is critical to understand the role and regulation of GLP-1 produced within the α-cell, as this may reveal new therapeutic avenues to selectively enhance intra-islet GLP-1 production.

Our previous work has shown that treatment with the GLP-1R agonist, liraglutide, induces α-cell expression of PC1/3 to enable GLP-1 production in both mouse and human islets (25). This effect is lost when the GLP-1R is ablated from β-cells (25). We previously demonstrated that liraglutide stimulates islet GLP-1 expression while simultaneously downregulating islet 14-3-3-ζ expression, an effect that requires the presence of the β-cell GLP-1R (11). 14-3-3 proteins are molecular scaffolds that regulate a wide array of signaling cascades by interacting with kinases, receptors, and other phosphoproteins (26). Among these, the ζ isoform has emerged as an important regulator of β-cell function (26), yet its mechanistic contribution to islet function is unclear. Notably, 14-3-3-ζ itself does not directly signal onto α-cells to regulate GLP-1 production (11). However, the extent to which this regulatory pathway influences α-cell function within an intact islet, particularly under physiologically relevant conditions such as nutrient stimulation and metabolic stress and whether these effects differ by sex, remains undefined.

The onset, progression, and hormonal regulation of obesity and type 2 diabetes exhibit clear sex-dependent differences, with an estimated 17.7 million more men than women diagnosed with type 2 diabetes worldwide (27). These clinical disparities are paralleled by sex differences in islet biology that likely contribute to divergent metabolic physiology and disease susceptibility between males and females (28-30). Despite these well-recognized differences, the impact of sex on α-cell biology remains incompletely defined. Although α-cell GLP-1 production has been reported in both males and females (11), whether its regulation or functional contribution to islet hormone secretion is sex-dependent remains unknown. Therefore, we performed a series of studies in mouse models with alterations in β-cell 14-3-3-ζ and α-cell GLP-1 expression to test the hypothesis that β-cell 14-3-3-ζ and α-cell GLP-1 exert sex-dependent regulation of islet function. We found that α-cell GLP-1 ablation impaired β-cell compensation in response to high-fat diet-induced metabolic stress in male mice. β-cell 14-3-3-ζ ablation improved glucose tolerance during a mixed-meal tolerance test and increased α-cell number per islet in male mice. Further, β-cell 14-3-3-ζ ablation increased GLP-1 production and GLP-1 secretion in both sexes. Interestingly, α-cell numbers were higher in females compared to males in both mouse and human islets, and α-cell GLP-1 production was higher in female mice. Together, these findings establish β-cell 14-3-3-ζ as a molecular brake on α-cell GLP-1 production, thereby coordinating islet hormone secretion and contributing to glucose homeostasis. More broadly, these data identify important sex-dependent differences in α-cell abundance and endocrine heterogeneity.

## Materials and methods

### Animal studies

For all studies, mice were housed individually in temperature- and humidity-controlled conditions with a 14:10-hour light-dark cycle. Both male and female mice were used, and study groups were weight-matched at the start of dietary interventions. The experimental protocols were approved by the UC Davis Institutional Animal Care and Use Committee.

To assess the role of α-cell GLP-1 in insulin secretion under metabolic stress, we studied wild-type (WT) *Gcg*CreERT^−^  *Pcsk1*^fl/fl^ and *Gcg*CreERT^+^  *Pcsk1*^fl/fl^ (α-cell PC1/3 KO) male and female littermates. For line validation, a cohort of male and female WT and α-cell PC1/3 KO mice were orally gavaged with tamoxifen (200 mg/kg/day; Sigma, T5648) for 5 consecutive days at 8 weeks of age. All mice received tamoxifen such that this variable did not differ between groups. Mice were maintained on a 60% energy from high-fat diet (HFD; Envigo, TD.06414) for 11 weeks, after which pancreases were collected and analyzed by immunohistochemistry (Fig. 1A). In addition, protein lysates were prepared from islets collected from mice used in the studies shown in Figs. 1 and 2, as detailed below, and total GLP-1 was measured by enzyme-linked immunosorbent assay (ELISA) (Meso Scale Discovery, Rockville, MD; Fig. S1A (31)).

**Figure 1:**
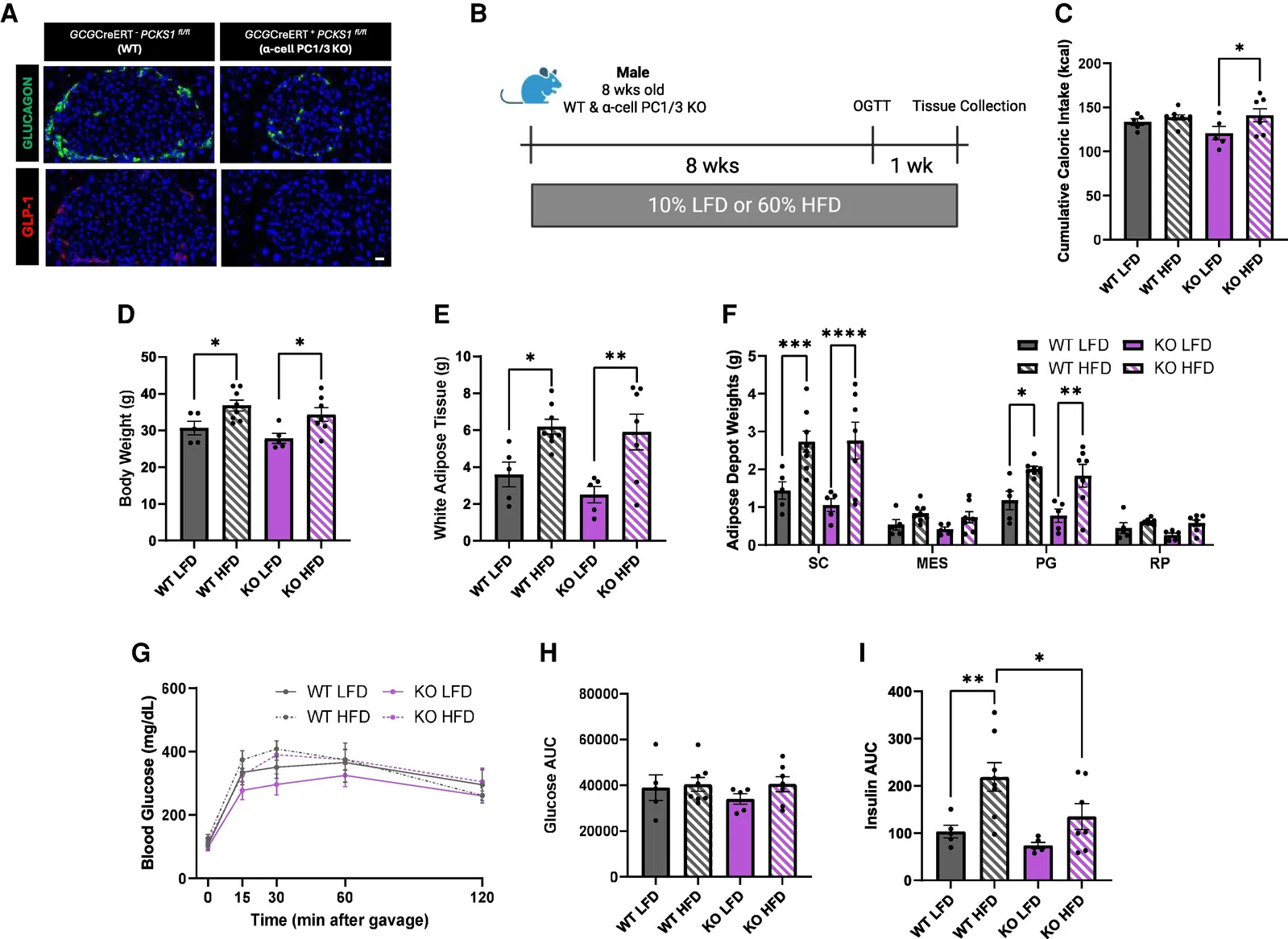
α-cell GLP-1 facilitates β-cell compensation in response to metabolic stress in male mice. (A) Representative images of mouse pancreas sections from *Gcg*CreERT^−^  *Pcsk1*  ^fl/fl^ (WT) and *Gcg*CreERT^+^  *Pcsk1*  ^fl/fl^ (α-cell PC1/3 KO) mice maintained on an HFD stained for glucagon, GLP-1, and DAPI. (B) Study design utilizing WT and α-cell PC1/3 KO male mice. (C) Cumulative caloric intake over an 11-day period on diet. (D) Body weight at the time of OGTT. (E) Total white adipose tissue (WAT) weight, and (F) subcutaneous (SC), mesenteric (MES), perigonadal (PG), and retroperitoneal (RP) adipose tissue weights. (G) Blood glucose concentrations, (H) glucose AUC (mg/dL × 120 minutes), and (I) serum insulin AUC (ng/mL × 60 minutes) during an OGTT. Data presented as mean ± SEM. *n* = 5 to 8 per group. *P < .05, **P < .01, ***P < .001, ****P < .0001 by 2-factor ANOVA with the Bonferroni posttest. Scale bar, 20 μm.

**Figure 2:**
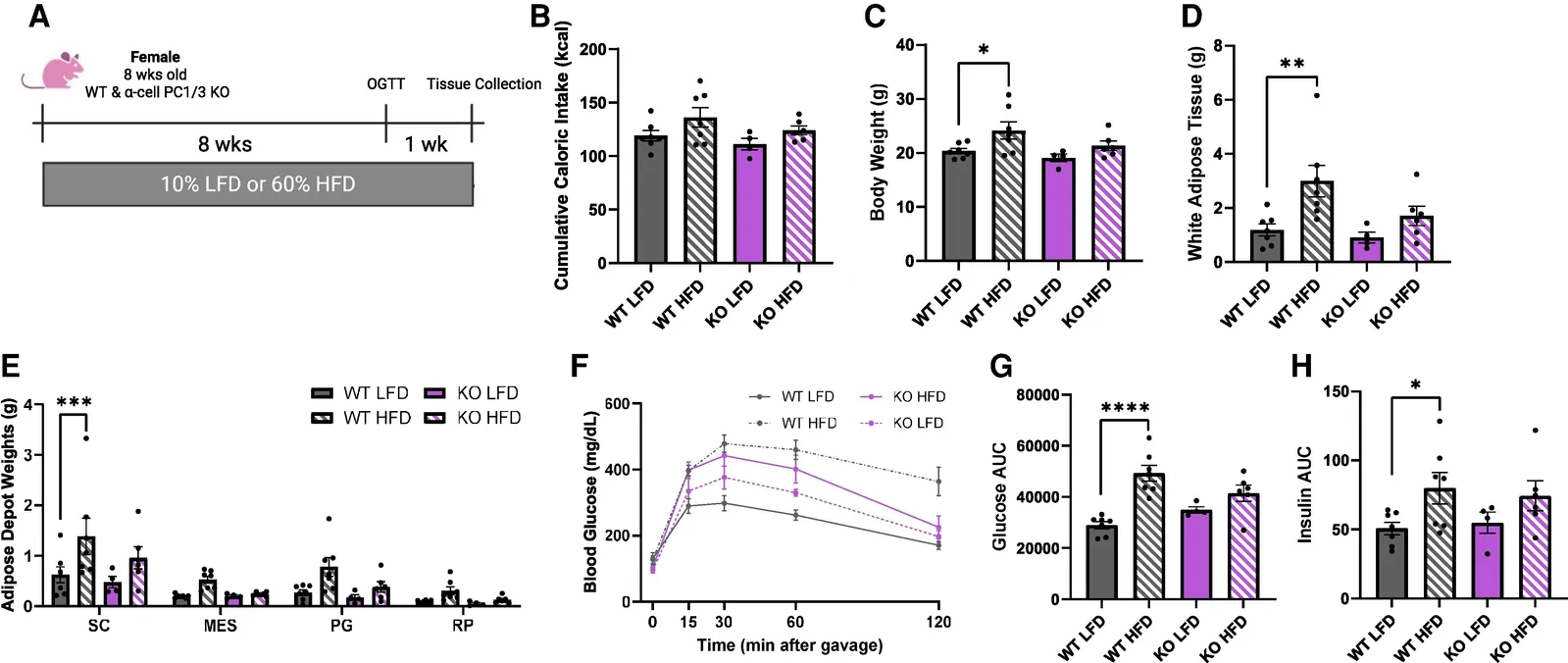
α-cell GLP-1 does not alter insulin secretion in female mice. (A) Study design utilizing female *Gcg*CreERT^−^  *Pcsk1*  ^fl/fl^ (WT) and *Gcg*CreERT^+^  *Pcsk1*  ^fl/fl^ (α-cell PC1/3 KO) mice studied with male mice in Fig. 1B. (B) Cumulative caloric intake over an 11-day period on diet. (C) Body weight at time of OGTT. (D) Total white adipose tissue (WAT) weight, and (E) subcutaneous (SC), mesenteric (MES), perigonadal (PG), and retroperitoneal (RP) adipose tissue weights. (F) Blood glucose concentrations, (G) glucose AUC (mg/dL × 120 minutes), and (H) serum insulin AUC (ng/mL × 60 minutes) during an OGTT. Data presented as mean ± SEM. *n* = 4-7 per group. *P < .05, **P < .01, ***P < .001, ****P < .0001 by 2-factor ANOVA with the Bonferroni posttest.

For data shown in Figs. 1 and 2, male and female WT and α-cell PC1/3 KO littermates were orally gavaged with tamoxifen (200 mg/kg/day) for 5 consecutive days starting at 8 weeks of age. All mice received tamoxifen such that this variable did not differ between groups. At 9 weeks of age, mice were placed on either a 10% energy from fat low-fat diet (LFD, Envigo, TD.08806) or 60% energy from fat HFD (Envigo, TD.06414). At 18 weeks of age, mice were fasted overnight, and an oral glucose tolerance test (OGTT) (2 g/kg of dextrose) was performed. Blood glucose was measured using a glucometer (One-Touch Ultra 2, Lifescan), and blood samples were collected from the tail. One week following the OGTT, mice were fasted overnight and killed by an overdose of pentobarbital (200 mg/kg of body weight, intraperitoneal), and tissues were weighed and collected. Serum insulin levels were quantified using ELISA (Millipore, Billerica, MA, EZRMI-13 K). Serum total GLP-1 concentrations were measured by ELISA.

To assess the impact of β-cell 14-3-3-ζ knockout in on glucose tolerance and insulin secretion, we studied WT *Ins1*Cre^−^  *14-3-3-ζ*  ^fl/fl^ and knockout *Ins1*Cre^+^  *14-3-3-ζ*  ^fl/fl^ (β-cell 14-3-3-ζ KO) male and female littermates (developed and previously validated (32)). Mice were enrolled at 8 weeks of age and maintained on either an LFD or HFD for 10 weeks. At 16 weeks of age, mice were fasted overnight, and an OGTT (2 g/kg of dextrose) was performed. Blood glucose recordings and serum were collected and analyzed as described above. Serum insulin (Millipore, Billerica, MA, EZRMI-13K) and glucagon (Mercodia, Salema, NC, 10-1281-01) concentrations were quantified using ELISA. Two weeks following the OGTT, mice were killed by an overdose of pentobarbital, and tissues were weighed and collected. In a separate cohort of mice, male and female WT and β-cell 14-3-3-ζ KO littermates were placed on an HFD at 8 weeks of age and maintained on the diet throughout the study. Body weight and food intake measurements were taken twice a week. Following 10 weeks on the diet, mice were fasted overnight and underwent a mixed meal tolerance test (200 μL of Ensure Plus supplemented with 30 mg of dextrose) via oral gavage (33). Two weeks following the mixed meal tolerance test (MMTT), mice were killed by an overdose of pentobarbital, and islets were isolated as described below; tissues were also weighed and collected.

To assess the impact of 14-3-3-ζ overexpression on the effect of liraglutide-induced insulin secretion, we studied whole-body WT and 14-3-3-ζ overexpressing (OE) male and female littermates (developed and previously validated (34)). Mice were enrolled at 8 weeks of age and maintained on an HFD for 10 weeks. Starting at 16 weeks of age, mice received twice-daily subcutaneous injections of either saline or liraglutide (200 μg/kg) for 2 weeks. Body weights were matched between groups at the start of liraglutide/saline treatment. After 2 weeks of liraglutide or saline treatment, mice were fasted overnight and given a last dose of liraglutide/saline 15 minutes before an OGTT (2 g/kg of dextrose). Blood glucose was measured as described above. Immediately following OGTT, mice were euthanized by an overdose of pentobarbital, and tissues were weighed and collected. Serum insulin levels were quantified by ELISA. Area under the curve (AUC) for blood glucose and serum insulin concentrations was calculated using the trapezoidal method. Insulin secretion index was calculated as InsulinAUC(0-120)/GlucoseAUC(0-120) during the OGTT, as previously described (35-39).

### Islet isolation and in vitro assays

Mouse islets were isolated from male and female mice after collagenase digestion, filtration, Histopaque gradient, and handpicking, as previously described (11). Islets were cultured overnight (37 °C, 5% CO_2_) in RPMI 1640 media with 16.1 mM glucose, 1% antibiotic–antimycotic, and 10% (v/v) fetal bovine serum. Following the overnight rest, islets were serum-starved in RPMI 1640 with 2.8 mM glucose for 2 hours. For total GLP-1 quantification, islets were handpicked into 6-well culture plates in 3 mL of media (10 islets per well). Islets were incubated in RPMI 1640 with either 2.8 mM or 16.1 mM glucose for 15 minutes before the collection of conditioned media. For active GLP-1 quantification, islets were handpicked into 48-well culture plates into 250 uL of media (10 islets per well). Islets were incubated in RPMI 1640 with an additional 2 mM of glutamine in either 2.8 mM or 16.1 mM glucose for 15 minutes before the collection of conditioned media. Total and active GLP-1 were measured by ELISA (MSD). Proteins were extracted using radioimmunoprecipitation assay buffer [10 mM tris-HCl (pH 7.4), 150 mM NaCl, 0.1% SDS, 1% Triton X-100, 1% sodium deoxycholate, 1 mM NaF, and protease inhibitors] and quantified with the bicinchoninic acid protein assay kit (Pierce, Thermo Fisher Scientific, Waltham, MA).

### qPCR

Total RNA was isolated and converted into complementary DNA (cDNA) with the iScript cDNA Synthesis Kit (Bio-Rad, Hercules, CA), as previously described (11). Gene expression was assessed by quantitative polymerase chain reaction using SYBR Green detection, and threshold values were normalized to *β-actin* for islet samples and *Gapdh* for brain samples. Primer sequences were as follows: *β-actin* 5′–3′ CAACGAGCGGTTCCGAT, 3′–5′ GCCACAGGATTCCATACCCA; *Glpr* 5′–3′ CGGCGTCAACTTTCTTATCTTCA, 3′–5′ GGGCGTGTTCGTCCA TCA; *Gcgr* 5′–3′ ATTGGCGATGACCTCAGTGTGA, 3′–5′ GCAATAGTTGGCTATGATGCCG; *Pcsk1* 5′–3′ CTCTGGTGGATTTGGCTGAT, 3′–5′ GGGCTCTAGGCTCAAAGTTATT; and *Gapdh* 5′–3′ CAATGTGTCCGTCGTGGA, 3′–5′ GATGCCTGCTTCACCACC.

### IHC and image analysis

Pancreas sections from mice studied as described above were further analyzed by immunohistochemistry (IHC), as previously described (25, 40, 41). In brief, pancreas samples were fixed in 4% paraformaldehyde and paraffin-embedded. Sections were deparaffinized in a xylene ethanol series and placed in Tris-EDTA (TE) buffer for antigen retrieval (10 mM Tris, 1 mM EDTA, 0.05% Tween, pH = 9.0) and then blocked with 9% normal goat serum and 1% bovine serum albumin (BSA). Mouse pancreas sections were immunostained for GLP-1 using a monoclonal antimouse amide antibody that has been extensively validated to recognize the amidated C-terminus of the GLP-1(7-36) peptide without cross-reactivity with nonamidated GLP-1, GLP-2, or glucagon (1:100; ab26278, Abcam, Cambridge, MA) (23, 42). Sections were stained for glucagon using a monoclonal anti-mouse antibody (1:100, G2654; Sigma Aldrich, St. Louis, MO) or a monoclonal anti-rabbit antibody (1:100, 27602; Sigma Aldrich) (Fig. 1A). Sections were stained for PC2 using a monoclonal rabbit antibody (1:1000,14013T; Cell Signaling, Danvers, MA) or Ki67 using a polyclonal rabbit antibody (1:100, ab15580, Abcam). Detection of primary antibodies was performed with Alexa Fluor 488 antirabbit (A11034) and Alexa Fluor 633 anti-mouse (A21052) secondary antibodies (1:200; Invitrogen, Foster City, CA). Nuclei were detected with 4′,6′-diamidino-2-phenylindole (P36962, Invitrogen). Pancreas image quantification and analysis were performed on all islets in a single longitudinal cross section of the pancreas, as previously described (25) (1 cross section per mouse; 10 islets per mouse on average for PC2, GLP-1, Ki67 and all detectable islets per mouse for glucagon). Islet imaging and quantification were performed in a blinded fashion. Islets were manually traced and quantified using ImageJ software. Islets were imaged using a Leica DM4 B fluorescent microscope with the Leica DFC7000 T color camera.

### Human pancreas analysis program

The PancDB database of human islet donors was accessed on 1/29/2026 (43, 44). Data were restricted to adults (≥18 years of age) and Healthy (“BMI < 30” and “No Diabetes”). No restrictions were applied for race, HbA1c, or antibody status. Donors listed as “pending” were excluded due to unavailable data. For each donor, islet cell counts (α, β, δ, ε, and γ cells) were normalized to the total number of islet cells to calculate the sex-specific percentage abundance of each cell type.

### Statistical analysis

Data are presented as mean ± SEM. All statistical analyses were performed using GraphPad Prism 10.00. Data were analyzed by 2-factor ANOVA with the Bonferroni posttest or Student *t*-test, as indicated. Differences were considered significant at *P* ≤ .05.

## Results

### α-cell GLP-1 facilitates β-cell compensation in response to metabolic stress

We previously demonstrated that α-cell ablation impairs liraglutide-induced insulin secretion in response to an intraperitoneal glucose challenge in male, but not female, mice (11). Therefore, we sought to determine the role of α-cell GLP-1 in the regulation of insulin secretion under healthy and metabolically stressed conditions. To address this, we generated a mouse model that enables inducible α-cell-specific *Pcsk1* knockout (α-cell PC1/3 KO; *Gcg*CreERT *Pcsk1*^fl/fl^). We crossed the *Gcg*CreERT (45) mouse line with a floxed *Pcsk1* mouse line (46). α-cell PC1/3 KO mice demonstrate a near-complete loss of α-cell GLP-1 production compared with WT mice (Fig. 1A and Fig. S1A (31)). While *Gcg*CreERT will knockdown PC1/3 in intestinal L cells, this only temporarily disables GLP-1 production in intestinal L cells, as these cells quickly turnover and are replaced by new GLP-1-expressing cells within 2 to 3 weeks (47). Consistent with this, both fasting and postprandial serum total GLP-1 concentrations were comparable between genotypes (Fig. S1B (31)). Furthermore, although preproglucagon neurons within the nucleus tractus solitarius represent the primary source of endogenous GLP-1 in the brain (48), *Pcsk1* mRNA expression in this region was unchanged between WT and α-cell PC1/3 KO mice (Fig. S1C (31)). Male WT and α-cell PC1/3 KO littermates were placed on an LFD or HFD at 9 weeks of age for 8 weeks (Fig. 1B). HFD-feeding increased caloric intake compared to LFD, but this only reached significance in α-cell PC1/3 KO male mice (Fig. 1C, *P* < .05). HFD feeding increased body weight and adiposity in both WT and α-cell PC1/3 KO male mice compared to LFD-fed controls (Fig. 1D-1F, *P* < .05), demonstrating that α-cell PC1/3 knockout does not alter susceptibility to diet-induced weight gain and adiposity in male mice. Glucose tolerance did not differ between diet or genotype during an OGTT (Fig. 1G, 1H); however, α-cell PC1/3 KO impaired β-cell functional adaptation to metabolic stress in male mice. Specifically, HFD feeding increased insulin secretion compared to LFD-fed controls in WT mice, but not in α-cell PC1/3 KO male mice. Further, α-cell PC1/3 knockout reduced insulin secretion compared to WT control male mice under HFD-fed conditions (Fig. 1I, *P* < .05).

Female WT and α-cell PC1/3 KO littermates were studied in parallel using the same paradigm as the male mice in Fig. 1B (Fig. 2A). Caloric intake did not differ between diet or genotype in female mice (Fig. 2B). However, HFD-feeding increased body weight and adiposity in WT but not α-cell PC1/3 KO female mice (Fig. 2C-2E, *P* < .05). Glucose tolerance was impaired, and insulin secretion was increased by HFD feeding in WT, but not α-cell PC1/3 KO female mice (Fig. 2F-2H, *P* < .05). However, insulin secretion did not differ between α-cell PC1/3 knockout and WT female mice under HFD-fed conditions (Fig. 2H). Overall, the blunted insulin secretion response in HFD-fed KO mice supports a role for α-cell-derived GLP-1 in supporting β-cell compensation during metabolic stress in male mice.

### β-cell 14-3-3-ζ ablation improves glucose tolerance in response to a mixed meal

We previously demonstrated that pan 14-3-3 inhibition enhances GSIS and activates α-cell GLP-1 production and secretion in isolated mouse and human islets, and that β-cell-specific deletion of 14-3-3-ζ similarly augments GSIS in chow-fed mice (11, 32). However, whether loss of β-cell 14-3-3-ζ is sufficient to improve glucose regulation under metabolic stress in vivo has not been examined. Therefore, we tested the hypothesis that ablation of 14-3-3-ζ in β-cells improves *in vivo* glucose regulation under HFD-induced metabolic stress. To test this, we studied male and female *Ins1*Cre^−^  *14-3-3-ζ*  ^fl/fl^ (WT) and *Ins1*Cre^+^  *14-3-3-ζ*  ^fl/fl^ (β-cell 14-3-3-ζ KO) mice (32). Male WT and KO littermates started receiving either an LFD or HFD at 8 weeks of age for 10 weeks (Fig. 3A). HFD feeding tended to increase caloric intake compared to LFD, but this only reached significance in β-cell 14-3-3-ζ KO male mice (Fig. 3B, *P* < .05). However, HFD feeding increased body weight and adiposity in both WT and β-cell 14-3-3-ζ KO male mice compared to LFD-fed controls (Fig. 3C–3E, *P* < .05), suggesting that β-cell 14-3-3-ζ ablation does not alter susceptibility to diet-induced weight gain and adiposity. HFD feeding impaired glucose tolerance during an oral glucose tolerance test compared to LFD-fed controls in WT but not β-cell 14-3-3-ζ KO male mice (Fig. 3F–3G, *P* < .05). β-cell 14-3-3-ζ ablation increased insulin secretion in HFD compared to LFD-fed male mice, whereas insulin secretion did not differ between WT LFD compared with HFD-fed male mice (Fig. 3H, *P* < .05). Fasting glucagon concentrations did not differ between groups (Fig. S2A (31)). Expression levels of the GLP-1 receptor (GLP1R) and glucagon receptor (GCGR) were comparable between WT and β-cell 14-3-3-ζ KO islets under HFD conditions, indicating that the effect of β-cell 14-3-3-ζ ablation to increase insulin secretion is not due to increased expression of receptors known to mediate α-cell potentiation of GSIS (Fig. 3I).

**Figure 3:**
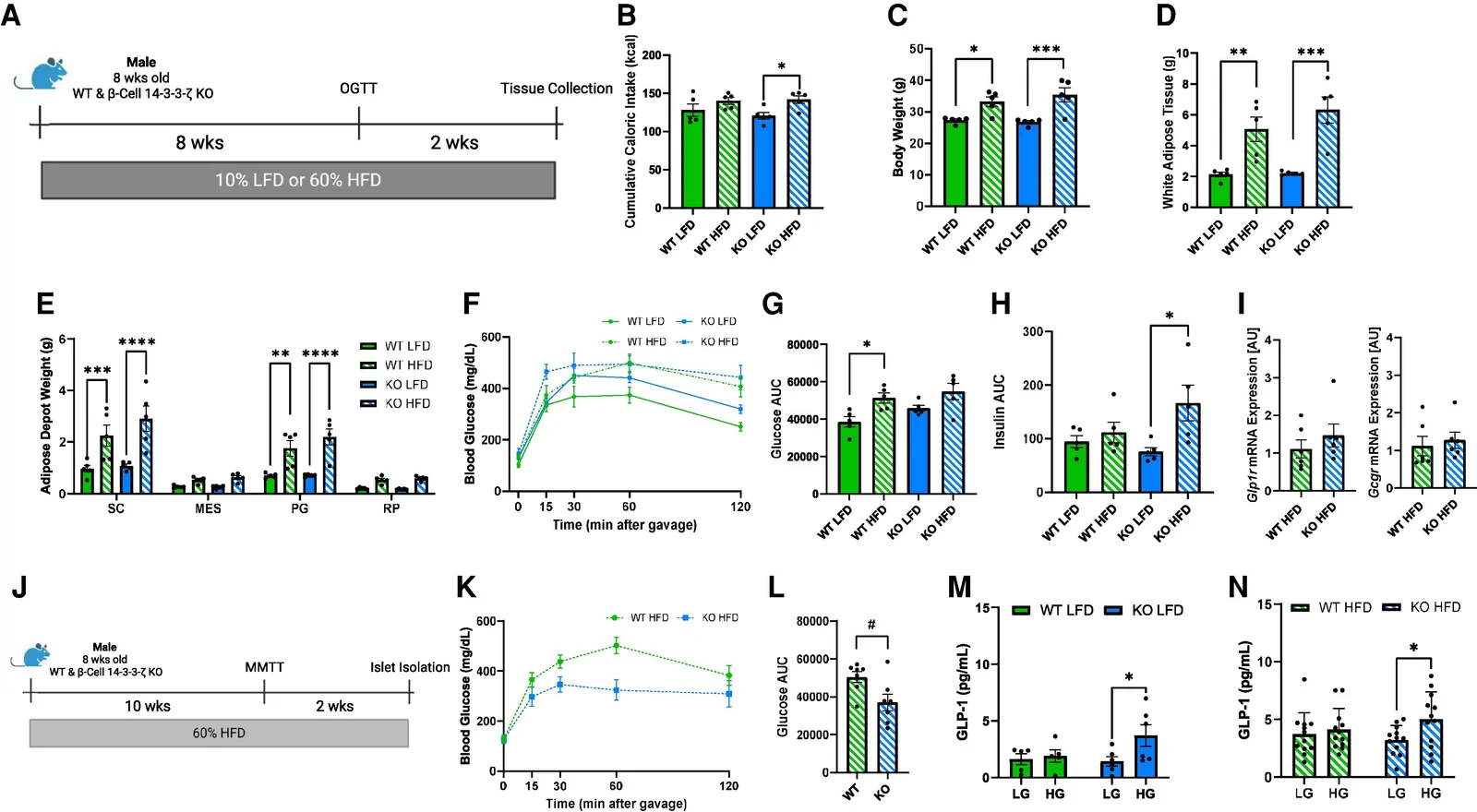
β-cell 14-3-3-ζ ablation improves glucose tolerance in response to a mixed meal in HFD-fed male mice. (A) Study design utilizing male *Ins1*Cre^−^  *14-3-3-ζ*  ^fl/fl^ (WT) and *Ins1*Cre^+^  *14-3-3-ζ*  ^fl/fl^ (β-cell 14-3-3-ζ KO) mice. (B) Cumulative caloric intake over a 12 day period on diet. (C) Body weight at time of euthanasia. (D) Total white adipose tissue (WAT) weight, (E) subcutaneous (SC), mesenteric (MES), perigonadal (PG), and retroperitoneal (RP) adipose tissue weights. (F) Blood glucose concentration, (G) glucose area under the curve (AUC, mg/dL × 120 minutes), and (H) serum insulin AUC (ng/mL × 60 minutes) during an OGTT. *n* = 5 per group. (I) Whole islet *Glp1r* and *Gcgr* mRNA expression from a separate cohort WT and β-cell 14-3-3-ζ KO HFD-fed mice*. n* = 6 per group. (J) Study design utilizing male mice from the same mouse model. (K) Blood glucose concentration, and (L) glucose AUC (mg/dL × 120 minutes) during a MMTT. *n* = 7 per group. (M) Total GLP-1 secretion from islets isolated from a separate cohort of WT and β-cell 14-3-3-ζ KO mice maintained on a chow diet under low glucose (LG, 2.8 mM) and high glucose (HG, 16.1 mM) conditions. *n* = 5-6 per group. (N) Active GLP-1 secretion from islets isolated from mice maintained on HFD studied in Fig. 3J. *n* = 12 per group. Data presented as mean ± SEM. *P < .05, **P < .01, ***P < .001, ****P < .0001 by 2-factor ANOVA with the Bonferroni posttest; ^#^*P* < .05 by the Student *t*-test.

To determine the impact of β-cell 14-3-3-ζ ablation on the glycemic response to a meal containing all 3 macronutrients, we conducted a MMTT in a separate cohort of WT and β-cell 14-3-3-ζ KO littermates maintained on an HFD (Fig. 3J). MMTTs include amino acids and lipids in addition to carbohydrates, offering a physiologically relevant stimulus for evaluating metabolic regulation. Similar to the prior cohort of mice, caloric intake, body weight, and adiposity did not differ between genotypes in either sex (Figs. S2 and S3 (31)). β-cell 14-3-3-ζ ablation improved glucose tolerance during an MMTT in male mice compared to WT controls (Fig. 3K, 3L*, P* < .05). The effect of β-cell 14-3-3-ζ ablation to improve glucose tolerance during an MMTT but not an OGTT may point to a role for the α-cell in glycemic regulation by 14-3-3-ζ, as amino acids are a key postprandial α-cell secretagogue. Therefore, we next evaluated the impact of β-cell 14-3-3-ζ ablation on α-cell GLP-1 secretion in isolated islets. High glucose (16.1 mM) stimulation for 15 minutes increased GLP-1 secretion compared to low glucose (2.8 mM) from islets isolated from chow-fed and HFD-fed β-cell 14-3-3-ζ KO mice but not in islets isolated from WT littermates (Fig. 3M, 3N, *P* < .05).

Female WT and β-cell 14-3-3-ζ KO littermates were studied in parallel using the same paradigm as the male mice in Fig. 3A (Fig. 4A). Similar to male mice, caloric intake increased in response to HFD feeding in β-cell 14-3-3-ζ KO female mice (Fig. 4B, *P* < .05). However, HFD feeding did not have an effect on body weight or total white adipose tissue weight in both WT and β-cell 14-3-3-ζ KO female groups (Fig. 4C, 4D). HFD-feeding increased subcutaneous adipose depot weight compared to LFD-fed control mice in the β-cell 14-3-3-ζ KO female mice (Fig. 4E, *P* < .05). HFD feeding did not impair glucose tolerance compared to LFD feeding in both genotypes (Fig. 4F, 4G). However, in contrast to male mice, β-cell 14-3-3-ζ KO HFD-fed female mice exhibited reduced insulin secretion compared to WT HFD controls (Fig. 4H, *P* < .05). Fasting glucagon concentrations were higher in WT LFD females than in WT HFD and β-cell 14-3-3-ζ KO LFD counterparts (Fig. S3A (31)). Additionally, glucose tolerance did not differ between WT and KO female mice during the MMTT (Fig. 4I-4K). These findings are consistent with our prior work finding no impact of β-cell 14-3-3-ζ ablation on insulin secretion in female mice (32). Collectively, these findings demonstrate that β-cell 14-3-3-ζ ablation improves glucose tolerance in male but not female mice following a mixed meal challenge.

**Figure 4:**
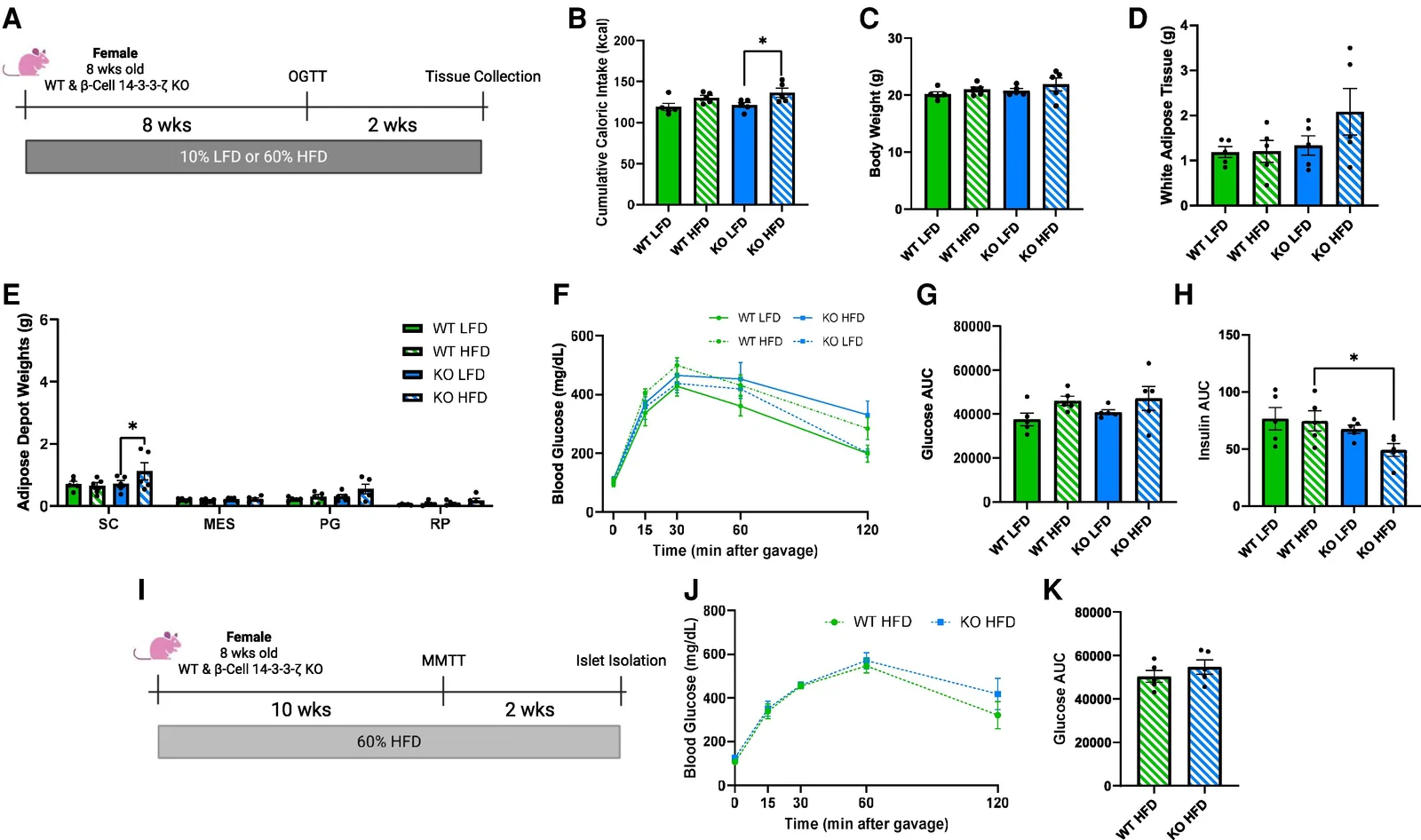
β-cell 14-3-3-ζ ablation does not improve glucose tolerance in response to a mixed meal in female mice. (A) Study design utilizing female *Ins1*Cre^−^  *14-3-3-ζ*  ^fl/fl^ (WT) and *Ins1*Cre^+^  *14-3-3-ζ*  ^fl/fl^ (β-cell 14-3-3-ζ KO) mice studied in parallel with male mice in Fig. 3A. (B) Cumulative caloric intake over a 12 day period on diet. (C) Body weight on the day of euthanasia. (D) Total white adipose tissue (WAT) weight, (E) subcutaneous (SC), mesenteric (MES), perigonadal (PG), and retroperitoneal (RP) adipose tissue weights. (F) Blood glucose concentration, (G) glucose area under the curve (AUC, mg/dL × 120 minutes), and (H) serum insulin AUC (ng/mL × 60 minutes) during an OGTT. (I) Study design utilizing female mice from the same mouse model. (J) Blood glucose concentration and (K) glucose AUC (mg/dL × 120 minutes) during a MMTT. Data presented as mean ± SEM. *n* = 5 per group. *P < .05 by 2-factor ANOVA with the Bonferroni posttest.

To complement these data, we tested whether 14-3-3-ζ overexpression alters the insulinotropic response to GLP-1R agonism. To this end, male and female WT and whole-body 14-3-3-ζ OE mice were placed on a HFD and treated with liraglutide (LIRA) or saline (CTRL) for 2 weeks (Figs. S4A and S5A (31)). In male mice, liraglutide treatment reduced caloric intake and improved glucose tolerance in both genotypes but increased insulin secretion only in WT mice (Fig. S4B–S4H (31)). Further, insulin secretion tended to be higher in liraglutide-treated WT compared with liraglutide-treated OE male mice (Fig. S4H (31), *P* = .06), suggesting that 14-3-3-ζ overexpression blunts the insulinotropic response to GLP-1R activation. While similar trends were observed in female mice, the effect of treatment and genotype often did not reach significance (Fig. S5 (31)). Of note, when data from both sexes were combined, liraglutide-treated WT mice exhibited significantly greater insulin secretion than liraglutide-treated OE mice (*P* < .05).

### β-cell 14-3-3-ζ ablation increases α-cell abundance and GLP-1 production

We next sought to determine the mechanisms by which β-cell 14-3-3-ζ ablation increases α-cell GLP-1 secretion. We assessed the impact of β-cell 14-3-3-ζ ablation on α-cell GLP-1 production in pancreatic sections collected from LFD- and HFD-fed WT and β-cell 14-3-3-ζ KO mice at 18 weeks of age (same mice studied in Fig. 3A). β-cell 14-3-3-ζ ablation increased the number of GLP-1^+^ α-cells per islet compared to WT control male mice under both HFD-fed and LFD-fed conditions (Fig. 5A, 5B, *P* < .001). Further, HFD increased α-cell GLP-1 production in WT male mice (Fig. 5A, 5B, *P* < .05). As α-cell GLP-1 production is often associated with inducers of α-cell proliferation (11, 25), we assessed Ki67 costaining with GLP-1. However, the number of Ki67^+^ GLP-1^+^ α-cells normalized to the total number of GLP-1^+^ α-cells per islet did not differ between groups (Fig. S6A, S6B (31)). Further, we tested whether the induction of α-cell GLP-1 production is associated with a decrease in PC2, the enzyme responsible for processing proglucagon to glucagon. The majority of GLP-1^+^ cells co-stained with PC2, and β-cell 14-3-3-ζ ablation increased the number of PC2^+^ GLP-1^+^ α-cells per islet under both LFD and HFD-fed conditions (Fig. 5C, *P* < .01). While PC2^−^ GLP-1^+^ α-cells were less abundant, HFD feeding increased the number of PC2^−^ GLP-1^+^ α-cells per islet in both genotypes (Fig. 5D, *P* < .05), suggesting that metabolic stress may promote a distinct GLP-1-producing α-cell state. Interestingly, β-cell 14-3-3-ζ ablation also increased the number of GCG^+^ α-cells per islet under both LFD and HFD conditions compared with WT controls in male mice (Fig. 5E, 5F, *P* < .05). However, the increase in α-cell abundance was not associated with a change in the number of Ki67^+^ GCG^+^ α-cells normalized to the total number of GCG^+^ α-cells per islet between groups (Fig. 5G).

**Figure 5:**
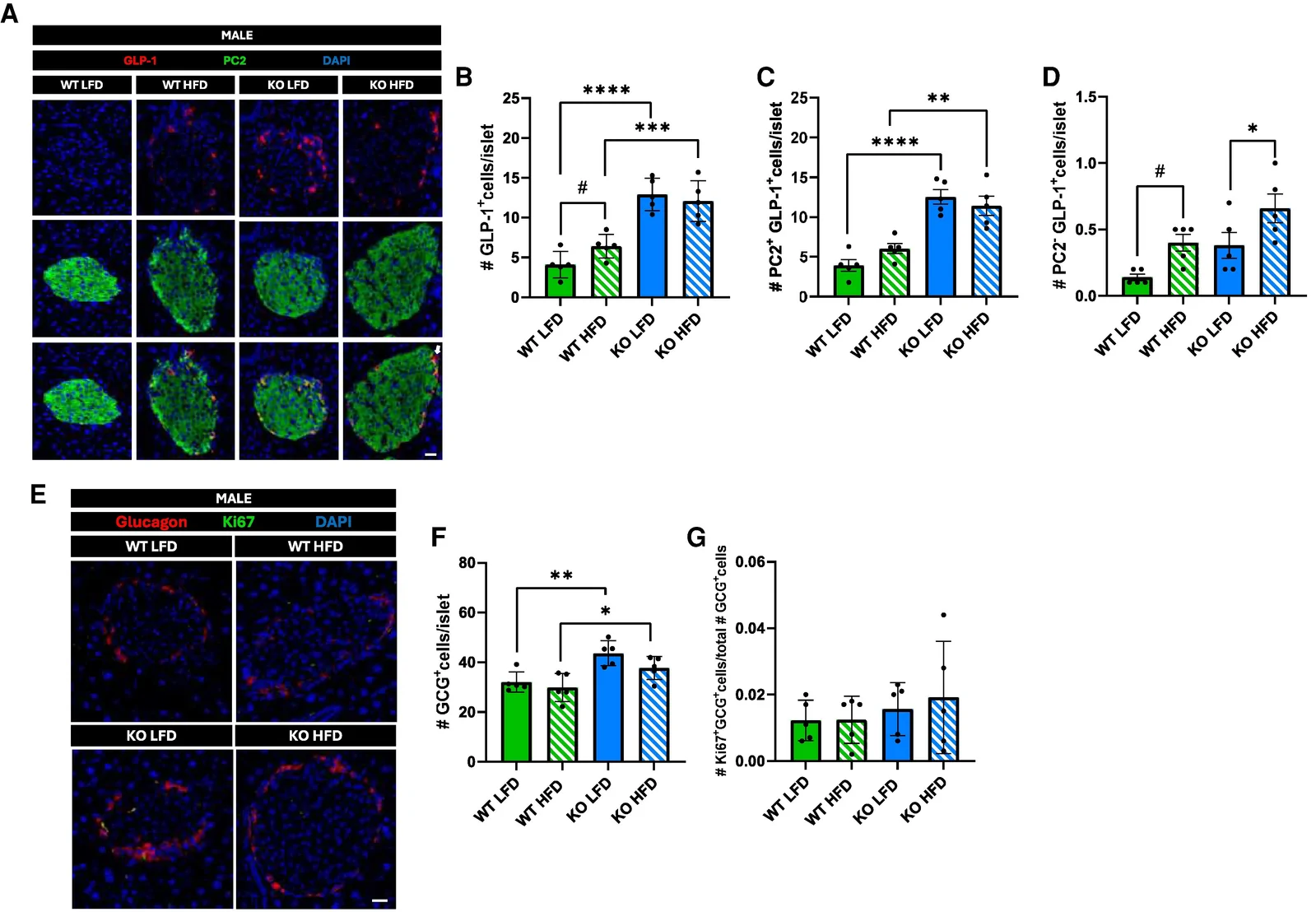
β-cell 14-3-3-ζ ablation increases α-cell abundance and GLP-1 production in male mice. (A) Representative images of mouse pancreas sections immunostained for GLP-1, PC2, and DAPI in male *Ins1*Cre^−^  *14-3-3-ζ*  ^fl/fl^ (WT) and *Ins1*Cre^+^  *14-3-3-ζ*  ^fl/fl^ (β-cell 14-3-3-ζ KO) mice studied in Fig. 3A. (B) Number of GLP-1^+^ cells per islet, (C) number of PC2^+^ GLP-1^+^ cells per islet, and (D) number of PC2^−^ GLP-1^+^ cells per islet. (E) Representative images of mouse pancreas sections immunostained for glucagon (red), Ki67 (green), and DAPI in male mice from the same study. (F) Number of GCG^+^ cells per islet, and (G) number of Ki67^+^ GCG^+^ cells normalized to the total number of GCG^+^ cells per islet. Data are presented as mean ± SEM. *n* = 5 per group. *P < .05, **P < .01, ***P < .001, ****P < .0001 by 2-factor ANOVA with the Bonferroni posttest; ^#^*P* < .05 by the Student *t*-test. Scale bar = 20 μm.

Female mice (same mice studied in Fig. 4A) displayed a similar increase in GLP-1^+^ α-cells per islet, as β-cell 14-3-3-ζ KO females had more GLP-1^+^ α-cells per islet compared to WT controls under both LFD- and HFD-fed conditions (Fig. 6A, 6B, *P* < .05). Most GLP-1^+^ cells costained with PC2, and were similarly elevated by β-cell 14-3-3-ζ ablation (Fig. 6C, *P* < .05). Similar to male mice, HFD feeding increased the number of PC2^−^ GLP-1^+^ α-cells per islet in WT female mice (Fig. 6D, *P* < .001). Further, β-cell 14-3-3-ζ ablation increased the number of PC2^−^ GLP-1^+^ α-cells per islet compared to WT controls under LFD, but not HFD, conditions (Fig. 6D, *P* < .001). As observed in males, the increase in GLP-1^+^ α-cells was not associated with a change in the number of Ki67^+^ GLP-1^+^ α-cells normalized to the total number of GLP-1^+^ α-cells per islet (Fig. S6C, S6D (31)). In contrast to male mice, the number of GCG^+^ α-cells per islet did not differ across study groups in female mice (Fig. 6E, 6F). Similar to male mice, the proportion of Ki67^+^ GCG^+^ cells relative to total GCG^+^ cells per islet did not differ between groups in female mice (Fig. 6G). Notably, across all female study groups, the number of Ki67^+^ GLP-1^+^ cells normalized to total GLP-1^+^ cells per islet was at least 2-fold higher than that of Ki67^+^ GCG^+^ cells (Fig. 6G and Fig. S6D (31)), reaching statistical significance within WT HFD and KO LFD groups (*P* < .05), suggesting that cell proliferation is associated with α-cell GLP-1 production.

**Figure 6:**
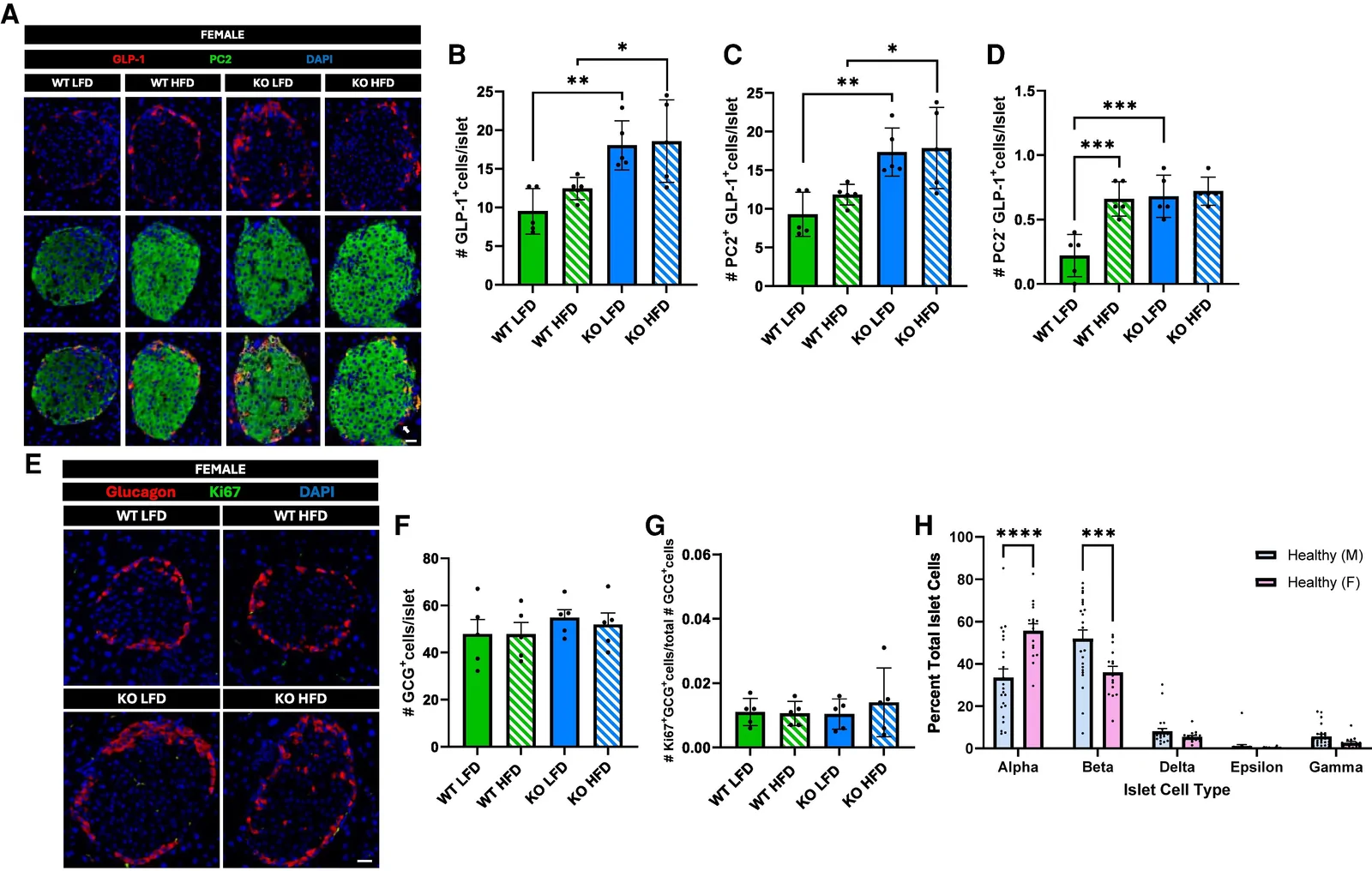
β-cell 14-3-3-ζ ablation increases α-cell GLP-1 production in female mice. (A) Representative images of mouse pancreas sections immunostained for GLP-1, PC2, and DAPI in female *Ins1*Cre^−^  *14-3-3-ζ*  ^fl/fl^ (WT) and *Ins1*Cre^+^  *14-3-3-ζ*  ^fl/fl^ (β-cell 14-3-3-ζ KO) mice studied in Fig. 4A. (B) Number of GLP-1^+^ cells per islet, (C) number of PC2^+^ GLP-1^+^ cells per islet, and (D) number of PC2^−^ GLP-1^+^ cells per islet. (E) Representative images of mouse pancreas sections immunostained for glucagon (red), Ki67 (green), and DAPI in female mice from the same study. (F) Number of GCG^+^ cells per islet, and (G) number of Ki67^+^ GCG^+^ cells normalized to the total number of GCG^+^ cells per islet. *n* = 5 per group. (H) Relative abundance of all islet cell types (percent of total islet cells) in islets from healthy human donors (defined as: BMI < 30 and not diabetic). Data from Human PancDB database. *n* = 16 to 23 per sex. Data presented as mean ± SEM. *P < .05, **P < .01, ***P < .001, ****P < .0001 by 2-factor ANOVA with the Bonferroni posttest. Scale bar = 20 μm.

Interestingly, female mice exhibited greater α-cell numbers per islet than males across all study groups except KO LFD (Tables 1 and 2). Further, female mice also had more GLP-1^+^ α-cells per islet than male mice across all conditions (Tables 1 and 2), supporting a sex-dependent enrichment of GLP-1-producing α-cells. To determine if the observed sex differences in α-cell numbers were translationally relevant, we used the Human Pancreas Database to determine islet cell abundance is healthy human subjects (43, 44). Among non-diabetic donors (BMI < 30), female donors exhibited a higher abundance of α-cells compared to male donors (Fig. 6H, *P* < .0001). Conversely, we found that male donors displayed a significantly higher abundance of β-cells (Fig. 6H, *P* < .001).

**Table 1:** Wild-type female mice have more GCG^+^ and GLP-1^+^ α-cells than male mice

	Male		Female			
	WT LFD	WT HFD	WT LFD	WT HFD	*P*-value M Vs F WT LFD	*P*-value M Vs F WT HFD
Total GCG^+^ cells/islet	32.0 ± 0.4	29.9 ± 0.5	47.9 ± 0.8	47.9 ± 0.7	.02	.01
Total GLP-1^+^ cells/islet	4.1 ± 0.3	6.4 ± 0.2	9.5 ± 0.3	12.4 ± 0.2	.02	.01

Number of total GCG^+^ cells and GLP-1^+^ cells per islet in *Ins1*Cre^−^  *14-3-3-ζ*  ^fl/fl^ (WT) male (M) vs female (F) mice fed a low-fat (LFD) or high-fat diet (HFD). Data presented as mean ± SEM and analyzed by 2-factor ANOVA with the Bonferroni posttest.

**Table 2:** β-cell 14-3-3-ζ KO female mice have more GLP-1^+^ α-cells than male mice and more GCG^+^ cells under conditions of metabolic stress only

	Male		Female			
	β-cell 14-3-3-ζ KO LFD	β-cell 14-3-3-ζ KO HFD	β-cell 14-3-3-ζ KO LFD	β-cell 14-3-3-ζ KO HFD	*P*-value M Vs F KO LFD	*P*-value M Vs F KO HFD
Total GCG^+^ cells/islet	43.7 ± 0.5	37.7 ± 0.4	54.8 ± 0.6	52.0 ± 0.7	.19	.05
Total GLP-1^+^ cells/islet	12.9 ± 0.3	12.1 ± 0.0	18.0 ± 0.4	18.6 ± 0.5	.03	.01

Number of total GCG^+^ cells and GLP-1^+^ cells per islet in *Ins1*Cre^+^  *14-3-3-ζ*  ^fl/fl^ (β-cell 14-3-3-ζ KO) male (M) vs female (F) mice fed a low-fat (LFD) or high-fat diet (HFD). Data are presented as mean ± SEM and analyzed by 2-factor ANOVA with the Bonferroni posttest.

Collectively, these findings show that β-cell 14-3-3-ζ ablation causes a sex-dependent increase in α-cell number per islet in male mice and increases the number of GLP-1^+^ α-cells per islet in a sex-independent manner. Further, we find that GLP-1^+^ α-cells are more proliferative than GCG^+^ α-cells. Finally, female mice have more α-cells per islet and more GLP-1^+^ α-cells per islet compared with male mice. Importantly, this sexually dimorphic increase in α-cell abundance is observed in human islets, supporting the concept that sex-specific islet composition and plasticity influence adaptive responses to metabolic stress.

## Discussion

This study identifies β-cell 14-3-3-ζ as a key regulator of intra-islet paracrine signaling that constrains α-cell GLP-1 production in vivo. Beyond establishing this regulatory axis, our findings demonstrate that islet architecture, α-cell abundance, and paracrine responsiveness are sexually dimorphic, revealing sex as a critical modifier of adaptive islet form and function under metabolic stress. Collectively, these data identify β-cell 14-3-3-ζ as an upstream constraint on α-cell GLP-1 production and reveal sex-dependent regulation of α-cell abundance, establishing β-cell 14-3-3-ζ and biological sex as key determinants of α-cell plasticity and islet GLP-1 production.

α-cells are heterogeneous in their endocrine profile, giving rise to functionally distinct subpopulations (11). Under conditions of metabolic stress, such as obesity and type 2 diabetes, α-cells oversecrete glucagon and exacerbate hyperglycemia (11, 16, 49, 50). Conversely, α-cells have also been shown to increase their active GLP-1 production under conditions of metabolic stress to help support β-cell function (2). However, what drives this shift in the α-cell endocrine profile remains poorly understood. Prior studies have demonstrated that α-cell GLP-1 can be induced by treating islets with streptozotocin (51), cytokines such as IL-6 (52), chemokines such as SDF-1 (53), and even high glucose (51). Given the divergent downstream pathways of these stimuli, the regulation of α-cell GLP-1 production is clearly complex and likely governed by multiple signaling pathways. Building on this concept and our previous work (11, 32), we now demonstrate that β-cell 14-3-3-ζ is an indirect regulator of α-cell GLP-1 production. Further, our findings build upon prior work demonstrating that α-cell-derived GLP-1 is required for β-cell adaptation during metabolic stress (2, 11).

We previously demonstrated that β-cell 14-3-3-ζ ablation increases GSIS in male mice and that islet 14-3-3-ζ expression is elevated in obesity and type 2 diabetes (32). Our findings suggest that the increase in β-cell 14-3-3-ζ expression in metabolic disease may impair β-cell activity indirectly by constraining α-cell GLP-1 production. Specifically, we find that α-cell GLP-1 ablation impairs β-cell compensation to HFD-induced metabolic disease. Further, we find that β-cell 14-3-3-ζ ablation increased α-cell GLP-1 production. Finally, we find that β-cell 14-3-3-ζ ablation improved glycemic response to a mixed meal but not to pure glucose challenge in male HFD-fed mice. The stronger glycemic improvements observed with the mixed meal tolerance test suggest that the benefits of 14-3-3-ζ ablation extend beyond direct effects on β-cell function and instead reflect altered intra-islet signaling dynamics in response to other nutrients, such as amino acids, known to stimulate α-cell hormone secretion postprandially.

Moreover, we identify a novel physiological role for β-cell 14-3-3-ζ in the regulation of α-cell biology, as β-cell 14-3-3-ζ ablation increased α-cell number per islet in male mice and the abundance of GLP-1^+^ α-cells in both male and female mice. While α-cell GLP-1 production is often associated with conditions involving an increase in α-cell numbers and/or proliferation (11), we did not observe a corresponding increase in Ki67 staining. It is possible that the proliferative window had passed by the time of analysis or that α-cell expansion occurred earlier in development due to the noninducible nature of the β-cell 14-3-3-ζ knockout. Nonetheless, these changes in islet structure and α-cell functionality were observed even under metabolically neutral conditions, suggesting that β-cell 14-3-3-ζ inherently constrains α-cell numbers and GLP-1 output in a healthy state. Of note, the effect of β-cell 14-3-3-ζ ablation to increase α-cell numbers may also promote enhanced insulin secretion through the action of glucagon; however, β-cell 14-3-3-ζ ablation did not increase circulating glucagon concentrations. Further, we identified an increase in a PC2^−^ GLP-1^+^ subpopulation of α-cells in response to β-cell 14-3-3-ζ in female mice and HFD feeding in male and female mice. α-cells are known to increase PC1/3 expression during obesity and diabetes (1-8, 17-23). However, it remains unclear whether this shift in PC1/3 expression alters PC2 expression. These data demonstrate that while the majority of GLP-1^+^ α-cells express PC2, it is possible to generate GLP-1^+^ α-cells that do not express the machinery needed to produce glucagon.

Our data also demonstrate clear sex-dependent differences in the role of β-cell 14-3-3-ζ in the regulation of glycemia and α-cell function. The effect of β-cell 14-3-3-ζ ablation to increase insulin secretion, improve glucose tolerance in response to a mixed meal, and increase α-cell number per islet was observed in male but not in female mice. Similarly, we find that α-cell-derived GLP-1 is required to facilitate β-cell compensatory responses to metabolic stress in male, but not female, mice. However, it is possible that the attenuated impact of β-cell 14-3-3-ζ and α-cell PC1/3 ablation in female mice may be due to their relative resistance to HFD-induced obesity compared to male mice. Indeed, female α-cell GLP-1 KO and WT and β-cell 14-3-3-ζ knockout mice did not differ in body weight between the LFD- and HFD-fed conditions. However, there were also important similarities between the effect of β-cell 14-3-3-ζ ablation in male and female mice. In particular, the effect of β-cell 14-3-3-ζ ablation to increase α-cell GLP-1 production was present in both sexes.

These studies also identify several important sex-dependent differences in islet morphology with a broader impact on our understanding of α-cell biology. First, we found that female mice have more α-cells per islet than males, independent of genotype. This is in line with prior studies finding higher α-cell area and mass relative to β-cell mass in female compared with male mice (54, 55). Notably, we find that this sex-dependent difference in α-cell abundance is also evident in healthy human islets. These observations point to possible developmental differences between sexes, potentially shaped by sex hormone signaling. Estrogen receptors are expressed in rodent and human islets (56-60), and estrogen signaling has been implicated in regulating metabolic health in both species (61-64). Moreover, disruption of estrogen signaling can alter mouse fetal pancreatic development by disrupting islet architecture, specifically the number and localization of glucagon-expressing cells (65). Second, we find that female mice have more GLP-1^+^ α-cells per islet than male mice. Consistent with this finding, prior work shows that estradiol stimulates GLP-1 production in mouse and human L cells and α-cells (60). Third, we find that GLP-1^+^ α-cells exhibited higher Ki67 colocalization compared with GCG^+^ α-cells in female mice. This finding is consistent with our previous work in which we found that human α-cells with increased *PCSK1* expression exhibited increased expression of other transcriptional markers of α-cell immaturity (25) and suggest that α-cell proliferation may contribute to α-cell GLP-1 production. Overall, further work is needed to understand the mechanisms driving sex-dependent differences in α-cell biology and how this influences sex-dependent differences in metabolic health.

Taken together, we identify β-cell 14-3-3-ζ as a molecular brake on α-cell GLP-1 production. Moreover, delineating distinct sex-dependent features of this β-to-α-cell axis uncovers previously underappreciated differences in α-cell abundance, GLP-1^+^ α-cell abundance, and islet function between males and females. These insights not only expand the mechanistic framework for α-to-β-cell communication but also identify β-cell 14-3-3-ζ as a potential therapeutic target for enhancing islet adaptability in a sex-informed manner. Collectively, this work identifies β-cell 14-3-3-ζ as an upstream regulator of α-cell GLP-1 production and demonstrates that this pathway operates differently between sexes.

## Data Availability

All datasets generated during and/or analyzed during the current study are not publicly available but are available from the corresponding author on reasonable request.

## Study approval

All animal studies were conducted in accordance with protocols approved by the Institutional Animal Care and Use Committee at the University of California, Davis.
